# Evaluation of rapid antigen detection kits for detection of SARS-CoV-2 B.1.617.2 virus

**DOI:** 10.2217/fvl-2021-0229

**Published:** 2022-04-11

**Authors:** Gannon CK Mak, Stephen SY Lau, Kitty KY Wong, Nancy LS Chow, Chi-Shan Lau, Edman TK Lam, Ken HL Ng, Rickjason CW Chan

**Affiliations:** ^1^Microbiology Division, Public Health Laboratory Services Branch, Centre for Health Protection, Department of Health, Hong Kong Special Administrative Region, China

**Keywords:** SARS-CoV-2, COVID-19, B.1.617.2, delta, rapid antigen detection

## Abstract

**Aim:** Currently, there is lack of data regarding rapid antigen detection (RAD) kits to detect SARS-CoV-2 B.1.617.2 virus. **Objective:** The purpose of this evaluation is to assess analytical sensitivity of 12 RAD kits against SARS-CoV-2 B.1.617.2. **Study design:** Analytical sensitivity was determined by limit of detection (LOD). A serial tenfold dilution set from a respiratory specimen collected from a COVID-19 patient infected by SARS-CoV-2 B.1.617.2 was used. RT-PCR was used as a reference method. **Results:** The LOD results showed that 11 and one RAD kits were 100- and 1000-fold less sensitive than RT-PCR respectively. **Conclusion:** The results showed that the RAD kits evaluated in this study may be used for first-line screening of the SARS-CoV-2 B.1.617.2 variant.

The gold standard to detect SARS-CoV-2 is RT-PCR. However, it takes hours to detect the nucleic acid from RNA extraction, amplification to results interpretation. In addition, specialized instrument and expertise are required. Rapid antigen detection (RAD) kits are alternative to RT-PCR due to the fast results and are easy to use, although these kits are inferior to RT-PCR in terms of sensitivity [1].

RAD kits detect viral protein by the immobilized coated SARS-CoV-2 antibody on the device. Data on specificity for the currently available RAD kits were consistently reported to be high [2]. The main disadvantages of this assay are the lack of amplification steps. Therefore, sensitivity of RAD kits cannot be comparable to RT-PCR. In addition, the performance of different RAD kits varied between different brands [3–8].

SARS-CoV-2 is continuously evolving. WHO classified SARS-CoV-2 as variants of concern (VOCs) for those having global public health impacts. At the time of writing this report, there are four VOCs, namely, B.1.1.7, B.1.351, P.1 and B.1.617.2, according to the PANGO nomenclature system [9–11]. SARS-CoV-2 B.1.617.2 is particularly important due to its high transmissibility. Moreover, this VOC is dominating worldwide when comparing with the other three VOCs [12–14]. In Hong Kong, due to the stringent containment strategies implemented [15], the daily number of COVID-19 cases remained to be low from May 2021 onwards and majority of them were limited to the imported cases [16] which was in contrast to 2020 [17,18]. To track variants, there is an intensive surveillance system which can detect VOCs circulating worldwide. Although rare, the author's system was capable of detecting local community acquired VOCs [19,20].

VOCs may affect the effectiveness of different diagnostic assays [21]. The possibility of impacts on RAD kits have been assessed for VOCs. Overall, most RAD kits can detect VOCs, minority differences were observed for some VOCs in terms of sensitivity [22,23]. For the recently emerged SARS-CoV-2 B.1.617.2, there is scant data on the parallel comparison between RAD kits. The purpose of this evaluation is to assess analytical sensitivity of RAD kits against SARS-CoV-2 B.1.617.2.

## Materials & methods

### SARS-CoV-2 B.1.617.2 virus

The Public Health Laboratory Services Branch (PHLSB) in Hong Kong has been designated as WHO COVID-19 reference laboratory since April 2020 and all confirmed cases in Hong Kong were either diagnosed or confirmed by PHLSB [24]. The leftover of the respiratory specimens after RNA extraction were stored at -70°C. From 7 January to 27 June 2021, PHLSB detected 77 SARS-CoV-2 B.1.617.2 cases in Hong Kong [20]. A respiratory specimen, combined nasopharyngeal and throat swabs, obtained from a COVID-19 patient collected on 12 May 2021 (hCoV-19/Hong Kong/VM21025593/2021) was selected for this evaluation. This specimen had sufficient quantity (>500 μl) and high viral load (Ct <20) which fulfil the criteria for this evaluation.

### RAD kits for SARS-CoV-2 detection

We routinely reviewed and evaluated RAD kits that were introduced to the authors' lab by local suppliers [7,8,25–27]. A total of 12 RAD kits were selected. Prior to this evaluation, we performed a screening and found that all of them shared similar analytical performance when tested against the SARS-CoV-2 culture isolate, the first SARS-CoV-2 case detected in Hong Kong (data not shown). The nucleotide sequence showed that it shared 99.97% identity with the strain WIV04 (GISAID accession number EPI_ISL_402124) detected in Wuhan, China, in late 2019 [28]. The nucleocapid (N) protein was 100% identical to WIV04. The details for each kit was summarized in Table 1. For the ease of communication, these kits were coded arbitrary from N01 to N12. N01–N06 were procured by the authors. The remaining six kits, N07–N12, were introduced to the author's lab by local suppliers during the period from December 2020 to June 2021 and were gifted by them for this evaluation. N01 and N02 were under the ‘WHO Emergency Use listing for *in vitro* diagnostics detecting SARS-CoV-2’ [29].

**Table 1.** Rapid antigen detection kits evaluated in the present study.

Kit	Manufacturer	Country of manufacturer	Code^‡^	Sample type	Target	Turnaround time
Roche SARS-CoV-2 Rapid Antigen Test^†^	SD BIOSENSOR	Republic of Korea	N01	Nasopharyngeal swab, specimens in transport media	Nucleocapsid	15–30 mins
Panbio COVID-19 Ag Rapid Test Device^†^	Abbott Rapid Diagnostics Jena GmbH	Germany	N02	Nasopharyngeal swab	Not specified	15–20 mins
INDICAID COVID-19 Rapid Antigen Test	PHASE Scientific International Ltd	Hong Kong SAR	N03	Nasal swab, nasopharyngeal swab	Not specified	20–25 mins
Aegle Coronavirus Ag Rapid Test Cassette	Zhejiang Orient Gene Biotech Co., Ltd	China	N04	Nasopharyngeal swab	Nucleocapsid	15–20 mins
BIOSYNEX COVID-19 Ag BSS	BIOSYNEX SWISS SA	Switzerland	N05	Nasopharyngeal swab	Nucleocapsid	15–20 mins
CLINITEST Rapid COVID-19 Antigen Test	Healgen Scientific Limited Liability Company	USA	N06	Nasopharyngeal swab	Nucleocapsid	15–20 mins
COVID-19 Antigen Test Kit	Nantong Diagnos Biotechnology Co., Ltd	China	N07	Nasal swab, throat swab	Nucleocapsid	10–15 mins
Rapid SARS-CoV-2 Antigen Test Card	MP Biomedicals Germany GmbH	Germany	N08	Nasal swab, nasopharyngeal swab, oropharyngeal swab	Nucleocapsid	15–20 mins
SARS-CoV-2 Virus Antigen Detection Kit	BGI PathoGenesis Pharmaceutical Technology Co., Ltd	China	N09	Nasal swab	Not specified	15–20 mins
AUISET COVID-19 Antigen Rapid Test Kit – New Saliva Test	Beijing Kewei Clinical Diagnostic Reagent Inc.	China	N10	Saliva secretion, oropharyngeal secretion	Nucleocapsid	15–20 mins
Rapid SARS-COV-2 Antigen Test Card	Ximan Boson Biotech Co., Ltd	China	N11	Nasopharyngeal swab	Nucleocapsid	15–20 mins
COVID-19 Antigen Saliva Test	ulti med Products (Deutschland) GmbH	Germany	N12	Saliva	Nucleocapsid	10 mins

This table was prepared by retrieving information from the manufacturer inserts delivered together with the kits.

† WHO Emergency Use listing for *in vitro* diagnostics detecting SARS-CoV-2.

‡ For the ease of communication throughout the article, each kit was assigned a code from N01 to N12.

The kits selected were based on lateral flow principles. SARS-CoV-2 antibody was immobilized coated on the test cassettes which can detect viral antigen. The test results can be interpreted by the naked eye. The test results were assessed and read by two technicians. Results grading of the band intensity were based on a previous study [23]. In case of doubtful results, the third technician interpreted the test results.

### Assessing analytical sensitivity of RAD kits

The dilution set of the specimen mentioned above was used to determine analytical sensitivity by means of limit of detection (LOD). The sensitivity of different kits can be obtained by measuring the lowest concentration of the specimen. To prepare the dilution set, serial tenfold dilution was performed for the stock of the specimen using viral transport medium (VTM). The VTM was in-house prepared by the authors and was the same VTM distributed to public and private hospitals and clinics in Hong Kong for respiratory specimen collection. Previously, the author's results showed that there were no effect on the sensitivity of RAD kits when using either VTM or phosphate-buffered saline as diluent [27]. Each dilution point was aliquoted and stored at -70°C until further testing.

A modified sample processing method was employed to perform the RAD kits since the authors wanted to unify the input volume. Regarding the input volume, the authors previously found that RAD results were affected by the input volume. Two input volumes were evaluated, 100 and 350 μl. The result bands were more intense when using 350 μl specimen volume [25] and this sample volume was selected for accessing LOD in the present study. The operating procedures were performed according to the manufacturer’s instructions, except first by mixing 350 μl specimen volume with the kit’s extraction buffer/diluent. Only one replicate was performed for each dilution point due to limited samples and limited quantity of kits.

To note: there was a sponge to collect the specimen volume for N12. Upon completing the specimen collection, the sponge was inserted into the test cassette. The author's preliminary evaluated the two sample processing methods by: (1) applying 350 μl specimen volume onto the sponge; and (2) applying 350 μl specimen volume directly into the test cassette. Positive results could not be obtained for (1) even though applying highest viral load of samples. It seemed to be related to absorbent effect of the sponge, a larger volume of sample should be used. On the other hand, positive results could be obtained for (2); and this sample processing method was chosen to evaluate N12 in the present study.

Virus concentrations in each dilution were estimated from cycle threshold (Ct) value as described [7]. Duplicates were performed for each dilution point and Ct values shown were the mean of both runs.

## Results

The LOD results for RAD kits against SARS-CoV-2 B.1.617.2 were summarized in Table 2. All except one kit shared similar analytical sensitivity, with the lowest concentration at dilution point 10^-3^. The LOD for RT-PCR was 10^-5^ which was 100-fold more sensitive than the RAD kits. The LOD difference between RAD kits and RT-PCR was concordant to the author's previous studies when using respiratory specimens of SARS-CoV-2 non-VOC strains [8,25,26].

**Table 2.** Comparison of limit of detection for 12 rapid antigen detection kits to detect SARS-CoV-2 B.1.617.2 virus.

Dilution	RT-PCR	Rapid antigen detection kits											
		N01	N02	N03	N04	N05	N06	N07	N08	N09	N10	N11	N12
10^-2^	22.73	+++	+++	+++	+++	+++	+++	+++	+++	+++	++	+++	+^†^
10^-3^	25.93	+	+	+	+	+	+	+	+	+	-	+	++
10^-4^	29.57	-	-	-	-	-	-	-	-	-	-	-	-
10^-5^	32.50	-	-	-	-	-	-	-	-	-	-	-	-
10^-6^	-	ND	ND	ND	ND	ND	ND	ND	ND	ND	ND	ND	ND

† The cassette showed abnormal diffusion when the specimen was applied.

The details of each kit can be referred to in Table 1. The RT-PCR and rapid antigen detection results were based on testing the serial tenfold dilution of the respiratory specimen, combined nasopharyngeal and throat swabs, obtained from a COVID-19 patient collected on 12 May 2021 (hCoV-19/Hong Kong/VM21025593/2021).

+++: Strong positive; ++: Positive; +: Weak positive; -: negative; ND: Not done.

For N10, the LOD was 10^-2^. It was found that the volume of extraction buffer/diluent was the largest when comparing with other 11 kits. Around 2 ml of extraction buffer/diluent was used for N10. The decreased in sensitivity for N10 was seemed to be related to the volume of extraction buffer/diluent used and the dilution effect of the specimen.

## Discussion

In the present study, the results showed that different RAD kits were capable of detecting SARS-CoV-2 B.1.617.2 virus with similar analytical sensitivity.

The SARS-CoV-2 VOCs are characterized by mutations in the *S* gene which can be screened by different single-nucleotide polymorphism real-time RT-PCRs with probes targeting these mutations [19]. Most RAD kits target SARS-CoV-2 N protein. This trend was also consistent in the present study. Mutations in the N protein have also been found in SARS-CoV-2 VOCs [30] which may affect the performance of RAD kits. RAD kits have also been used in other viruses. In the author's previous findings, the reduced sensitivity of RAD in detecting influenza A(H1N1)pdm virus when comparing with seasonal A(H1N1) was most likely due to the mismatch of the swine virus origin antigen and the monoclonal antibody to the nucleoprotein of human viruses [31]. Unlike RT-PCR, the performance of different assays can be checked by aligning the sequences of primers and probes against SARS-CoV-2 viruses. Since antibodies used for RAD kits are unknown and checking the performance of RAD kits against different SARS-CoV-2 viruses was impossible. Recently, different reports showed that the performance of RAD kits were affected by the genetic background SARS-CoV-2 viruses or mutations in the N protein [32–34]. There is a lack of information whether other mutations in the N protein can affect RAD kits. The N protein of SARS-CoV-2 B.1.617.2 was characterized by three mutations, D63G, R203M and D377Y [30] when comparing with the reference strain WIV04. The author's previous sequencing results showed that the strain used in the present study also shared these three mutations, other mutations were not found [20]. The data highlighted that these three mutations did not affect the effectiveness of RAD kits evaluated in the present study.

The purpose of this study is to estimate if the commercially available RAD kits are suitable for diagnosing SARS-CoV-2 B.1.617.2 infection; however, accurately and precise ranking of RAD kits is not the objective. It is ideal to perform more dilution points such as 1:2 and 1:5 between the two serial tenfold dilution points. In addition, each dilution point should be performed in replicates. Given that extra quantity of specimens and RAD kits were required, on top of the already strained capacity of the laboratory, it was determined that a serial tenfold dilution and single replicate were to be adopted in the study design. According to the previous results, analytical sensitivity of different RAD kits can vary hugely when comparing with RT-PCR. When serial dilution of a respiratory specimen was used to assess LOD of RAD kits, some RAD kits can be 10^5^-fold less sensitive than RT-PCR while the best RAD kits can be only 10^2^-fold less sensitive than RT-PCR [7,8]. It means that a 1000-fold difference of analytical sensitivity of RAD kits can be obtained. A dilution set of serial tenfold dilution and single replicate could fulfil the objective. In the present study, the kits selected were either based on the author's prior evaluation experiences or products that recently emerged in the market. All except one kit shared the same LOD. The kit having one log lower than the others was mostly due to the diluent effect of the buffer used. It is encouraging to see that the performance of RAD kits improved as time goes on.

The limitations of this study include using only one SARS-CoV-2 B.1.617.2 virus to assess analytical sensitivity. The authors were incapable of using fresh samples for evaluation since viral load of different samples varied. The author's recent analysis showed the viral load of SARS-CoV-2 samples collected from local community varied widely, Ct values ranged from 16.28 to 36.94 [35]. The viral load of different samples have to be estimated by RT-PCR first. On the other hand, SARS-CoV-2 samples had to be investigated to ensure the correct VOC selected. Using a frozen stored sample was inevitable based on this study design. However, this issue was not a matter of concern. Since the same set of dilution was used to assess different RAD kits and a fair comparison could be performed. In addition, the present study focused on assessing analytical sensitivity of RAD kits. Although we did not use clinical specimens for evaluation, LOD results correlated well with clinical sensitivity according to the author's past studies [7,8,25,26]. In addition, the RAD kits evaluated in the present study detected lowest concentrations at Ct 25 which was in line with the recent review of summarizing 24 studies worldwide [36]. This review concluded that RAD kits were most sensitive for samples of Ct value ≤25. When there is a large demand of RAD kits, the LOD approach can provide a quick screening method. The results therefore showed that the RAD kits used in this study may be used for first-line screening of the SARS-CoV-2 B.1.617.2 variant. Finally, specificity of RAD kits was not tested for; however, the RAD kits demonstrated high specificity [2].

## Conclusion

It is expected that many RAD kits for diagnosing SARS-CoV-2 infection are emerging and also the types of SARS-CoV-2 variants. Different studies have been reported for the performance of RAD kits against different SARS-CoV-2 variants. These results showed that SARS-CoV-2 variants demonstrated from no, minor or significant effects on RAD kits [22,23,32–34]. The performance of RAD kits should be regularly monitored so that guidance can be provided to different clinical settings.

**Summary points**

- The analytical sensitivity of 12 rapid antigen detection kits against SARS-CoV-2 B.1.617.2 were evaluated.
- All except one kit were 100-fold less sensitive than RT-PCR.
- The remaining kit was 1000-fold less sensitive than RT-PCR, which may be due to the dilution effect of buffer/diluent used.
- The performance of rapid antigen detection kits should be regularly monitored.
